# Unique Outbreak of Rift Valley Fever in Sudan, 2019

**DOI:** 10.3201/eid2612.201599

**Published:** 2020-12

**Authors:** Ayman Ahmed, Yousif Ali, Adel Elduma, Mawahib Hassan Eldigail, Rehab Abdallah Mhmoud, Nouh Saad Mohamed, Thomas G. Ksiazek, Isabelle Dietrich, Scott C. Weaver

**Affiliations:** University of Khartoum, Khartoum, Sudan (A. Ahmed);; University of Texas Medical Branch, Galveston, Texas, USA (A. Ahmed, T.G. Ksiazek, S.C. Weaver);; World Reference Center for Emerging Viruses and Arboviruses, Galveston (A. Ahmed, S.C. Weaver);; Sudan Federal Ministry of Health, Khartoum (Y. Ali, A. Elduma, M.H. Eldigail);; River Nile State Ministry of Health, Eldamar, Sudan (R.A. Mhmoud);; Nile University, Khartoum (N.S. Mohamed);; The Pirbright Institute, Pirbright, UK (I. Dietrich)

**Keywords:** arboviral diseases, arboviruses, epidemic, epizootic, mosquito-borne diseases, One Health, outbreak, Rift Valley fever, RVF, Sudan, vectorborne diseases, zoonoses

## Abstract

We report a unique outbreak of Rift Valley fever in the Eldamar area, Sudan, May–July 2019, that resulted in 1,129 case-patients and 19 (1.7%) deaths. Patients exhibited clinical signs including fever (100%), headache (79%), and bleeding (4%). Most (98%) patients also reported death and abortions among their livestock.

Rift Valley fever (RVF) is an arboviral disease caused by RVF virus (RVFV; genus *Phlebovirus*, family *Phenuiviridae*) (*1*). RVFV periodically emerges to cause epizootics among livestock and epidemics in persons living nearby (*2*). It is mainly transmitted by the bite of infected mosquitoes or by direct contact with infected animals and their products (*3*). In addition, RVFV transmission is maintained vertically among both humans and vector mosquito populations (*4*,*5*). 

The public health threat from arboviral diseases is growing rapidly in Sudan (*4*,*6*). Increasing human movement, often arising from armed conflict, is driving several arboviral diseases to emerge in Sudan, usually in the form of undifferentiated febrile illness. Recent epidemics include dengue fever (*6*,*7*), Crimean-Congo hemorrhagic fever, West Nile virus disease (*8*), yellow fever, and chikungunya fever (*4*). RVF outbreaks represent major public health and economic burdens on endemic countries, particularly those such as Sudan that rely on exporting animals and animal products (*3*,*4*,*9*). In a recent study, RVFV infection was also associated with spontaneous abortion among pregnant women in Sudan (*10*). We describe a unique, undeclared outbreak of RVF in River Nile state, in northern Sudan, leading to the potential spread of the virus to other states in Sudan or neighboring countries. 

## The Study

**Figure 1:**
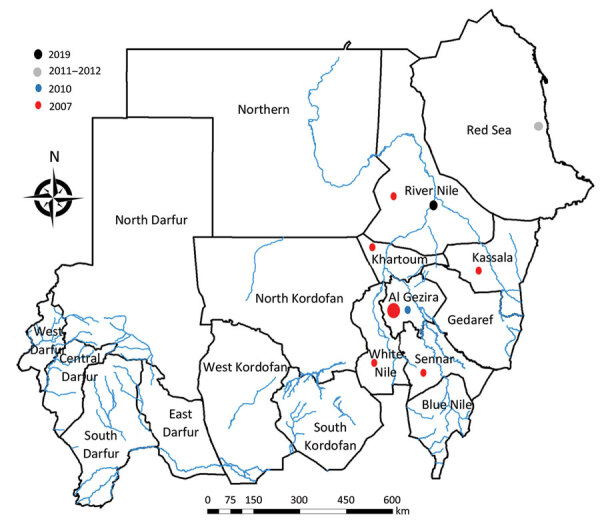
Distribution of Rift Valley fever outbreaks in Sudan, by year.

**Figure 2:**
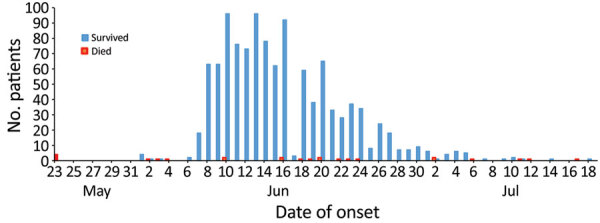
Cases of and deaths from Rift Valley fever, River Nile state, north Sudan, May 23–July 18, 2019.

Rift Valley fever cases initially appeared on May 23, 2019, in Eldamar, the capital city of River Nile state (Figure 1). This region, characterized as a desert environment, is in general rural and peri-urban; most of the population relies on farming, animal breeding, and more recently, traditional gold mining. By July 18, a total of 1,129 cases had been identified on the basis of clinical signs and symptoms. The outbreak peaked in June 2019 with »96 cases reported daily (Figure 2).

Most RVF cases, 1,120 (99.2%), were from Eldamar, with only 7 cases reported in Barbar and 2 in Atbara (Table). The male:female ratio was 1.2:1. Adults 25–44 years of age were most affected (34.6%), but other age groups were similarly represented; however, only 6.1% of cases involved children <5 years old (Table). Many of the patients (34%) worked in farming and animal product production. 

**Table:** Characteristics of case-patients during Rift Valley fever outbreak in Sudan, 2019

Characteristic	No. cases	% Of total
Sex		
F	505	45
M	624	55
Locality		
Eldamar	1,120	99.2
Barbar	7	0.6
Atbara	2	0.2
Age group, y		
<5	69	6.1
5–14	229	20.3
15–24	233	20.7
25–44	391	34.6
>44	207	183
Signs/symptoms		
Fever	1129	100
Headache	892	79
Bleeding	45	4
Reported disease/abortion among domestic animals		
Yes	1104	98
No	25	2

Among the 1,129 patients, 100% had fever, 79% (892/1,129) had headache, and only 4% (45/1,129) had clinically manifested bleeding. Almost all (98%; 1,104/1,129) patients (or their guardians, in the case of children) reported death or abortion among their domestic livestock. Of the 19 reported human deaths, 6 (32%) were children <15 years of age and 9 (47.4%) were farmers (Table). 

We retrospectively analyzed data collected by active surveillance during this epidemic to confirm that RVFV was the exclusive causative agent of this outbreak, because it is normally associated with the rainy season in Sudan. Surveillance was established by the River Nile State Ministry of Health. In our analysis, we included variables such as patient age and sex; signs and symptoms, such as fever, hemorrhagic manifestations, and headaches; where the patient lived; and if the patient had noted any death or abortion among livestock. We randomly collected 50 blood samples and tested them by reverse transcription PCR using the RealStar Rift Valley Fever Virus RT-PCR Kit 1.0 (Altona Diagnostics GmbH, https://altona-diagnostics.com) in the Sudanese National Public Health Laboratory, Khartoum, Sudan. PCR testing confirmed RVFV infection in 88% (44/50) of samples. 

We report a unique outbreak of RVF that occurred before the typical transmission season, which in Sudan normally corresponds with rain and flooding during September–December. There were also an unusually large number of cases (1,129) and 19 related deaths. Although RVF epidemics and epizootics are common in Sudan because of the endemic transmission of RVFV (*4*), this outbreak was unique in the scale of human and animal infections over only 3 months. The timing of outbreak development was also unusual, which could be attributed to the nationwide political violence that forced many people to move with their animals from RVF-endemic to non–RVF-endemic areas and vice versa (*11*). 

Three major RVF epidemics were documented in Sudan in 2007, 2010, and 2011–2012 (Figure 1) (*4*). During June 2011–November 2012, a total of 28 RVFV infections were detected among pregnant women in the governmental hospital of Port Sudan in Red Sea state (*10*). In 2010, the outbreak was more limited in size and geographic distribution; only 18 cases were reported, in El Gezira state (*12*). In 2007, a total of 747 human RVF cases, including 230 deaths, were reported from El Gezira, Sennar, White Nile, Kassala, Khartoum, and River Nile states; most cases (54%) and deaths (64%) were reported from El Gezira state (*9*). 

In Sudan, there is no reliable estimate for RVF death and abortion rates among livestock animals due to limited health surveillance systems for both human and animal populations (*4*,*9*,*13*). However, a 2019 report shared with the World Organization for Animal Health showed that, in sheep, the death rate was 2.4% and the case-fatality rate 13.5%; in goats, the death rate was 2.1% and the case-fatality rate 18.8% (*14*). 

As part of the recent unrest in Sudan, medical and health professionals have been intentionally targeted by military forces for their role in supporting national demonstrations. As a result, both health outcomes and records of cases might have been influenced by the escalating political violence throughout the country during the time of the 2019 RVF outbreak (*11*). These attacks impaired the health system, limiting its capacity to respond to and contain the outbreak in its early stages (*11*). This health system deficiency is underscored by the limited number of cases reported around June 16, 2019, when the outbreak was at its peak (Figure 1). In addition, Eldamar is a major market for domestic animals, particularly sheep and goats, which could have been an additional risk factor influencing the emergence of RVFV in the area and facilitating spread into other states, particularly Port Sudan in Red Sea state, the main seaport of the country for exporting animals (*15*). Unfortunately, Sudan typically experiences delays and deficiencies in data sharing during epidemics and health emergencies (*13*), which likely contributed to the slow reporting of this 2019 RVF outbreak and the general impairment of the health system during a time of national political change (*11*). 

## Conclusions

The risk of exporting RVFV across borders remains poorly characterized but real, stressing the need for a countrywide, One Health survey in Sudan to investigate the prevalence of RVFV among humans and domesticated animals and to identify the risk factors associated with its emergence. Further, we recommend vaccination of domestic animals at risk during the dry season when they cluster together before being moved to open pastures (*4*) and adherence by health authorities to the core of the World Health Organization’s International Health Regulations, including sharing data in a timely manner to promote both local and international safety (*13*). 

This major RFV outbreak underscores the urgent need in Sudan for improved surveillance systems and a robust health policy for the prevention, early detection, and control of arboviral epidemics. In the case of RVF, early reporting of information regarding animal health is particularly important, because it could be used to contain the disease before it spills over into people. 
